# Survival benefit after neoadjuvant or adjuvant radiotherapy for stage II–III gastroesophageal junction adenocarcinoma: A large population-based cohort study

**DOI:** 10.3389/fonc.2022.998101

**Published:** 2022-10-20

**Authors:** Zhichao Zuo, Yafeng Peng, Ying Zeng, Shanyue Lin, Weihua Zeng, Xiao Zhou, Yinjun Zhou, Bo Li, Jie Ma, Mingju Long, Shenghui Cao, Yang Liu

**Affiliations:** ^1^ Department of Radiology, Xiangtan Central Hospital, Xiangtan, China; ^2^ Department of Radiology, Affiliated Hospital of Guilin Medical University, Guilin, China; ^3^ Department of Radiology, Guangxi Medical University Cancer Hospital, Nanning, China; ^4^ Department of General Surgery, Xiangtan Central Hospital, Xiangtan, China; ^5^ Department of Radiotherapy, Guangxi Medical University Cancer Hospital, Nanning, China

**Keywords:** neoadjuvant radiotherapy, adjuvant radiotherapy, survival, gastric cancer, gastroesophageal junction adenocarcinoma (GEJA)

## Abstract

**Objective:**

The standard treatment for stage II–III gastroesophageal junction adenocarcinoma (GEJA) remains controversial, and the role of radiotherapy (RT) in stage II–III GEJA is unclear. Herein, we aimed to evaluate the prognosis of different RT sequences and identify potential candidates to undergo neoadjuvant RT (NART) or adjuvant RT (ART).

**Materials and methods:**

In total, we enrolled 3,492 patients with resectable stage II–III GEJA from the Surveillance, Epidemiology, and End Results (SEER) database, subsequently assigned to three categories: T_1–2_N^+^, T_3–4_N^−^, and T_3–4_N^+^. Survival curves were evaluated using the Kaplan–Meier method along with the log-rank test. We compared survival curves for NART, ART, and non-RT in the three categories. To further determine histological types impacting RT-associated survival, we proposed new categories by combining the tumor, node, and metastasis (TNM) stage with Lauren’s classification.

**Results:**

ART afforded a significant survival benefit in patients with T_1–2_N^+^ and T_3–4_N^+^ tumors. In addition, NART conferred a survival advantage in patients with T_3–4_N^+^ and T_3–4_ exhibiting the intestinal type. Notably, ART and NART were both valuable in patients with T_3–4_N^+^, although no significant differences between treatment regimens were noted.

**Conclusions:**

Both NART and ART can prolong the survival of patients with stage II–III GEJA. Nevertheless, the selection of NART or ART requires a concrete analysis based on the patient’s condition.

## Introduction

Previously, gastroesophageal junction adenocarcinoma (GEJA) and gastric cancer have been regarded as the same disease and treated using identical methods. Based on the current perspective, significant differences exist between GEJA and gastric cancer in terms of etiology and epidemiology. Therefore, it is necessary to examine GEJA as a distinct disease (1). Given the unique anatomical location of GEJA, the optimal treatment strategy remains controversial. Currently, surgery is considered the most effective treatment option for GEJA. However, patients with stage II and III GEJA reportedly exhibit poor survival rates with surgical intervention alone, and there is a high recurrence rate within 2 years, even after complete resection (2). Therefore, a perioperative treatment strategy is crucial for patients with GEJA, especially in advanced stages.

Based on the results of the Intergroup-0116 (INT-0116) trial conducted in the United States, adjuvant radiotherapy (ART) has become an important treatment strategy for resectable GEJA, and subgroup analysis revealed that individuals with Lauren’s classification-intestinal type were more likely to benefit from ART (3). However, the ARTIST and ARTIST-II trials performed in Korea have presented contrasting opinions, demonstrating that ART could not considerably reduce the incidence of recurrence post-D2 gastrectomy, which is also documented in the CRITICS trial (4–6). In addition, the subgroup analysis of ARTIST has afforded a similar result, indicating that ART failed to benefit patients with the intestinal type. Accordingly, the role of neoadjuvant radiotherapy (NART) in gastric cancer and GEJA has been explored. According to the findings of a meta-analysis, NART could improve the survival of patients with GEJA (7). This result has been supported by several randomized clinical trials (8–10). NART is a promising strategy to improve outcomes of advanced-stage GEJA. To provide additional data for clinical decision-making regarding the use of radiotherapy (RT) in advanced-stage GEJA, we compared the prognosis of different RT sequences and identified potential candidates for NART or ART.

## Materials and methods

### Patients

**Figure 1:**
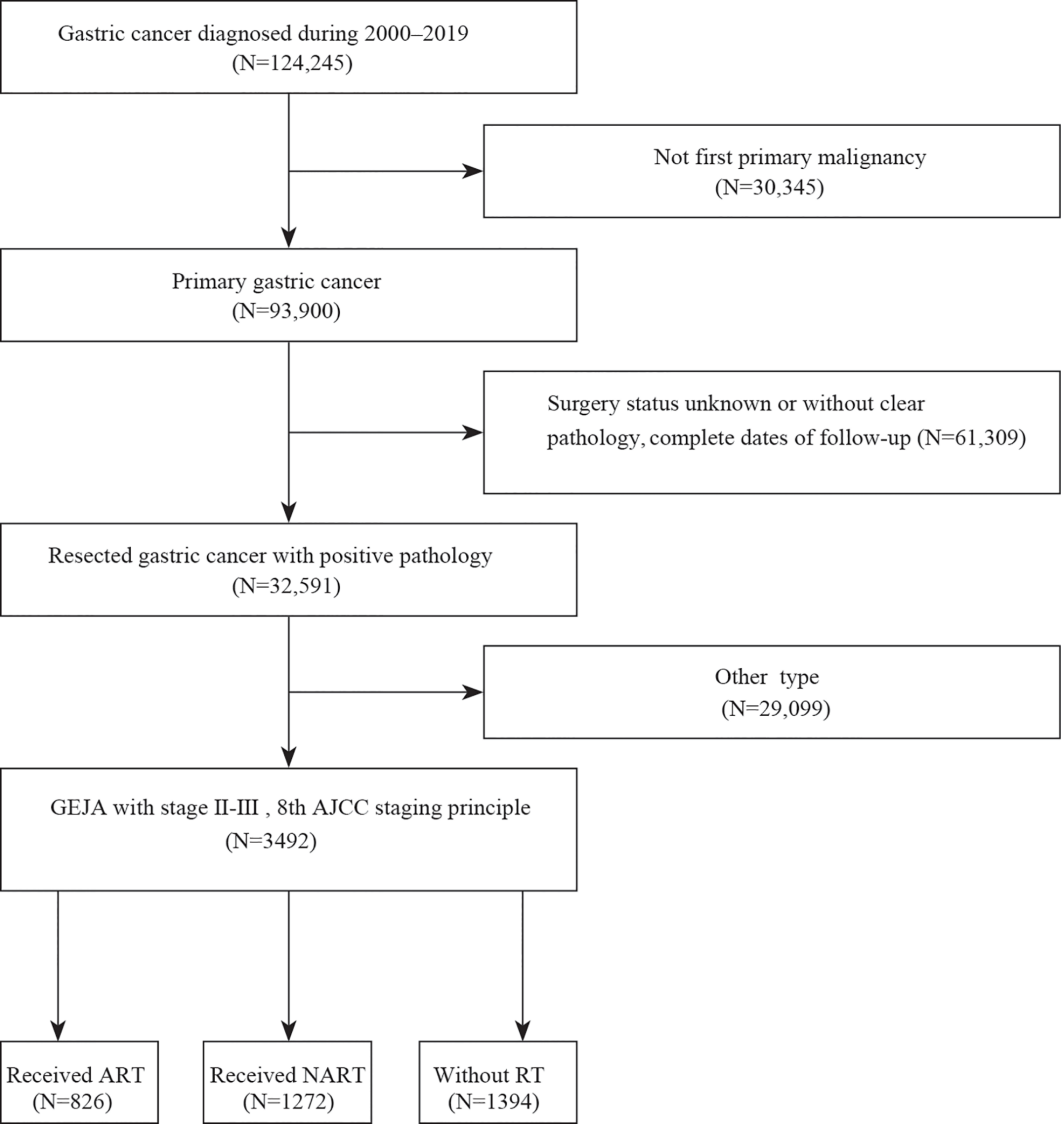
Study flowchart.

Data of patients diagnosed with GEJA were retrieved from the Surveillance, Epidemiology, and End Results (SEER) database (diagnosed during 2000–2019) using the SEER*Stat software (version 8.3.6). Herein, enrollment criteria were as follows: a) patients with first primary malignancy, b) patients who underwent surgery and were histologically diagnosed with stage II–III GEJA, c) reassessed pathologic tumor stage [based on the collaborative stage (CS) site information provided by the SEER database and the number of positive lymph nodes cleared], corresponding to the American Joint Committee on Cancer (AJCC) 8th TNM staging system for gastric cancer (11). The exclusion criteria were as follows: a) patients with metastasis, unclear pathological tumor stage, and missing information regarding lymph node status; b) patients with survival durations of less than 30 days; and c) patients who received pre- and postoperative RT. **Figure 1** presents a flowchart of the study.

### Study variables

We used the International Classification of Diseases for Oncology (ICD-O, third edition) to classify gastric adenocarcinoma according to Lauren’s classification, as follows: diffuse carcinoma (8,145), linitis plastica (8,142), and signet ring cell carcinoma (8,490); intestinal type includes adenocarcinoma, not otherwise specified (8,140), adenocarcinoma, intestinal type (8,144), adenocarcinoma, tubular (8,211), and carcinoma, not otherwise specified (8,010) (12).

We retrospectively reviewed the database to determine the demographic, clinical, and biological characteristics of enrolled patients. The main outcome variables were survival status and survival time. Survival time was measured as overall survival (OS), the period from the date of surgery to the date of death or the last follow-ups.

### Statistical analysis

Survival curves were evaluated using the Kaplan–Meier method along with the log-rank test. We assigned patients to three categories (T_1–2_N^+^, T_3–4_N^−^, and T_3–4_N^+^) based on the initial T or N status following the 8th AJCC staging principle; subsequently, we compared survival curves of NART, ART, and non-RT in the three patient categories. To further determine histological types that impact RT-associated survival, we proposed new categories by combining the TNM stage with Lauren’s classification. A univariate Cox regression analysis was conducted to evaluate prognostic factors, and multivariate Cox models were constructed and assessed using the survival and “pec” packages. In addition, decision curve analysis (DCA) was performed using the “DCA” package. The calibration curves were plotted using the “rms” package to assess the calibration of the model. All analyses were performed using R version 4.0.1 software (R Foundation for Statistical Computing, Vienna, Austria).

## Results

### Baseline characteristics of patients

Herein, we enrolled 3,492 patients with resectable stage II–III GEJA who received NART or ART. In total, 2,825 (80.9%) patients were male, with a median age of 65 years (range, 20–90 years). Among these patients, 2,098 (60.3%) received RT (826 received ART, and 1,272 received NART). Factors associated with the RT sequence are presented in **Table 1**, including sex, age, marital status, race, grade, histological type, surgery, number of examined lymph nodes (ELNs), T stage, N stage, and chemotherapy.

**Table 1:** Clinical and pathological features in patients with stage II–III gastroesophageal junction adenocarcinoma, stratified by RT sequence.

Characteristics	Total(N = 3,492)	ART(N = 826)	NART(N = 1,272)	Non-RT(N = 1,394)	*p-*Value
**Sex**					<0.001
Female	667 (19.1%)	171 (20.7%)	184 (14.5%)	312 (22.4%)	
Male	2,825 (80.9%)	655 (79.3%)	1,088 (85.5%)	1,082 (77.6%)	
**Age**					<0.001
≤65 years	1,739 (49.8%)	451 (54.6%)	742 (58.3%)	546 (39.2%)	
>65 years	1,753 (50.2%)	375 (45.4%)	530 (41.7%)	848 (60.8%)	
**Marital status**					<0.001
Married	2,408 (69.0%)	569 (68.9%)	919 (72.2%)	920 (66.0%)	
Single	390 (11.2%)	85 (10.3%)	150 (11.8%)	155 (11.1%)	
Others	694 (19.9%)	172 (20.8%)	203 (16.0%)	319 (22.9%)	
**Race**					<0.001
Black	155 (4.44%)	49 (5.93%)	33 (2.59%)	73 (5.24%)	
White	3,051 (87.4%)	687 (83.2%)	1,175 (92.4%)	1,189 (85.3%)	
Others	286 (8.19%)	90 (10.9%)	64 (5.03%)	132 (9.47%)	
**Grade**					<0.001
I/II	1,179 (33.8%)	255 (30.9%)	504 (39.6%)	420 (30.1%)	
III/IV	2,313 (66.2%)	571 (69.1%)	768 (60.4%)	974 (69.9%)	
**Histological type**					<0.001
Diffuse type	516 (14.8%)	152 (18.4%)	153 (12.0%)	211 (15.1%)	
Intestinal-type gastric adenocarcinoma	2,658 (76.1%)	595 (72.0%)	1,030 (81.0%)	1,033 (74.1%)	
Others	318 (9.11%)	79 (9.56%)	89 (7.00%)	150 (10.8%)	
**Surgery**					<0.001
Gastrectomy, NOS	2,584 (74.0%)	574 (69.5%)	1,020 (80.2%)	990 (71.0%)	
Near total/total gastrectomy	677 (19.4%)	198 (24.0%)	173 (13.6%)	306 (22.0%)	
Partial gastrectomy	231 (6.62%)	54 (6.54%)	79 (6.21%)	98 (7.03%)	
**ELNs**					<0.001
<15	1,569 (44.9%)	341 (41.3%)	666 (52.4%)	562 (40.3%)	
≥15	1,923 (55.1%)	485 (58.7%)	606 (47.6%)	832 (59.7%)	
**T stage**					<0.001
T1	66 (1.89%)	22 (2.66%)	13 (1.02%)	31 (2.22%)	
T2	1,042 (29.8%)	337 (40.8%)	188 (14.8%)	517 (37.1%)	
T3	1,518 (43.5%)	204 (24.7%)	839 (66.0%)	475 (34.1%)	
T4	866 (24.8%)	263 (31.8%)	232 (18.2%)	371 (26.6%)	
**N stage**					<0.001
N0	900 (25.8%)	72 (8.72%)	562 (44.2%)	266 (19.1%)	
N1	970 (27.8%)	227 (27.5%)	371 (29.2%)	372 (26.7%)	
N2	875 (25.1%)	262 (31.7%)	243 (19.1%)	370 (26.5%)	
N3	747 (21.4%)	265 (32.1%)	96 (7.55%)	386 (27.7%)	
**Chemotherapy**					<0.001
None	825 (23.6%)	60 (7.26%)	3 (0.24%)	762 (54.7%)	
Yes	2,667 (76.4%)	766 (92.7%)	1,269 (99.8%)	632 (45.3%)	


NART, neoadjuvant radiotherapy; RT, radiotherapy; ART, adjuvant radiotherapy; ELN, number of examined lymph node.

### Survival benefit after neoadjuvant radiotherapy or adjuvant radiotherapy in patients with TNM stage and intestinal subtype

**Figure 2:**
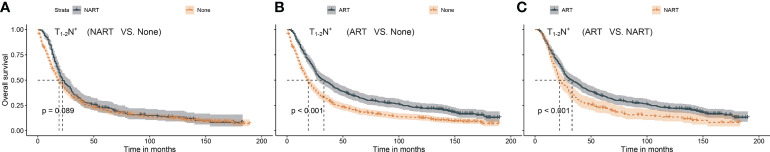
**(A–C)** Survival benefit after NART or ART in patients with T_1–2_N^+^. **(A)** NART failed to benefit patients with T_1–2_N^+^. **(B)** ART prolonged survival in patients with T_1–2_N^+^. **(C)** Overall survival after NART was worse than that after ART in patients with T_1–2_N^+^. NART, neoadjuvant radiotherapy; ART, adjuvant radiotherapy.

**Figure 3:**
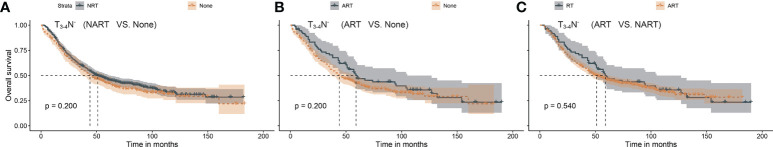
**(A–C)** Survival benefit after NART or ART in patients with T_3–4_N^−^. Patients with T_3–4_N^−^ had no survival advantage after any ART or NART. NART, neoadjuvant radiotherapy; ART, adjuvant radiotherapy.

**Figure 4:**
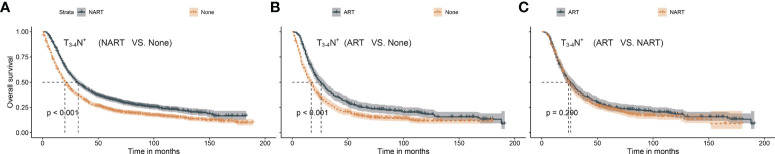
**(A–C)** Survival benefit after NART or ART in patients with T_3–4_N^+^. Survival benefit was obtained after both NART and ART but did not differ significantly between the two RT sequences. NART, neoadjuvant radiotherapy; ART, adjuvant radiotherapy.

In the present study, the median and maximum follow-up periods were 104 and 191 months, respectively. The 5-year overall survival rates were 31.3%, 33.4%, and 23.1% for ART, NART, and non-RT, respectively. Considering that the T stage and nodal status can impact RT in gastric cancer (13), the patients were assigned to three categories: T_1–2_N^+^, T_3–4_N^−^, and T_3–4_N^+^. Based on the survival analysis, ART (*p* < 0.001) but not NART (*p* = 0.089) could benefit patients with the T_1–2_N^+^ stage, as presented in **Figure 2**. As shown in **Figure 3**, both ART and NART failed to afford survival benefits in patients with T_3–4_N^−^ (both *p* = 0.200). As shown in **Figure 4**, both NART and ART conferred survival benefits in patients with T_3–4_N^+^ (both *p* < 0.001), although no significant differences were noted between the two RT sequences (*p* = 0.290).

**Figure 5:**
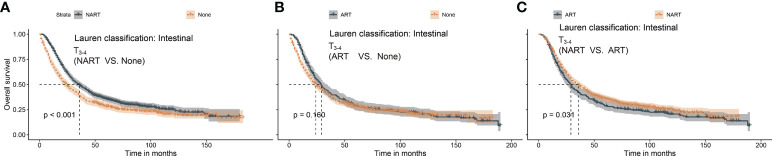
**(A–C)** Survival benefit after NART or ART in T_3–4_ patients with intestinal type. **(A)** NART prolonged survival in T_3–4_ patients with intestinal type. **(B)** ART failed to benefit survivors in T_3–4_ patients with intestinal type. **(C)** Overall survival after ART was worse than that after NART in T_3–4_ patients with intestinal type. NART, neoadjuvant radiotherapy; ART, adjuvant radiotherapy.

In summary, ART conferred significant survival benefits in patients with T_1–2_N^+^ and T_3–4_N^+^, whereas survival advantages after NART were only noted in patients with T_3–4_N^+^. In a previous report (14), patients with Lauren’s classification-intestinal type were found to exhibit a response to NART. Therefore, we proposed a new category based on the intestinal type of Lauren’s classification in patients with T_3–4_. As shown in **Figure 5**, patients with T_3–4_ presenting intestinal type benefited from NART (*p* < 0.001) rather than ART (*p* = 0.160).

### Prognostic factors in patients with resectable stage II–III gastroesophageal junction adenocarcinoma

**Figure 6:**
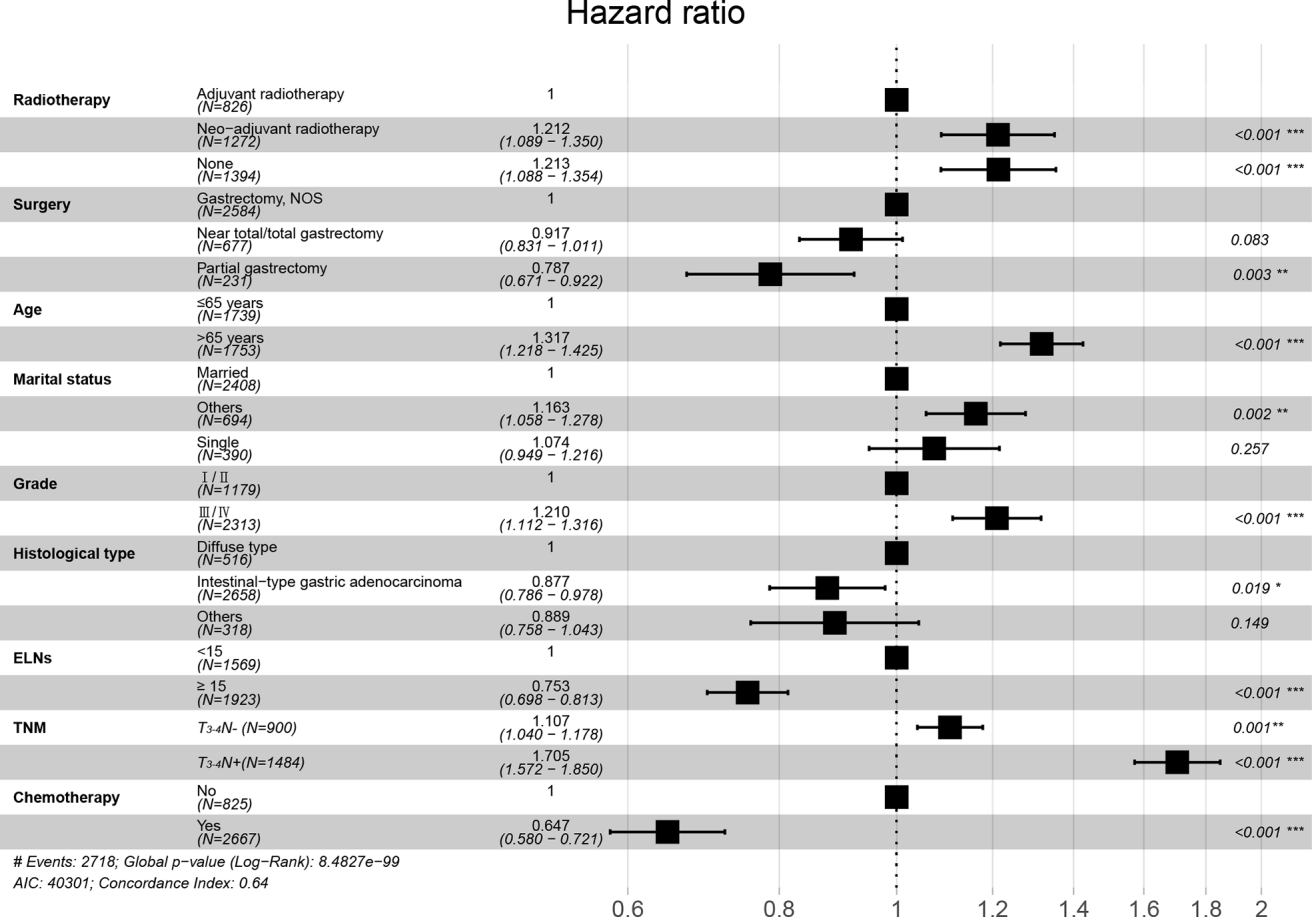
Forest plot of mortality rates.

**Figure 7:**
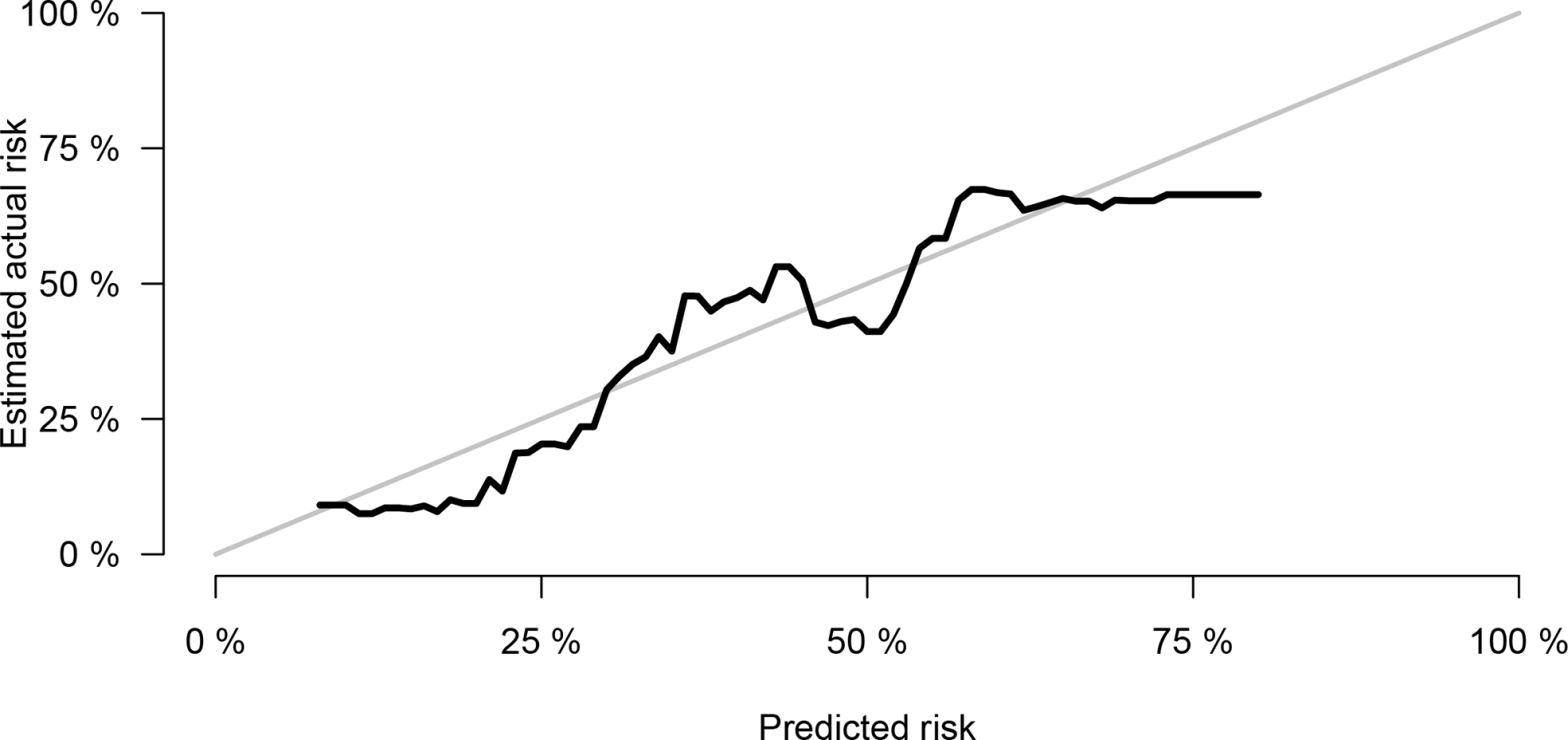
Decision curve analysis of Cox model.

The results of univariate Cox regression analysis revealed that several clinical and biological characteristics, such as RT sequence, age, tumor grade, histological type, surgery, ELNs, TNM stage, and chemotherapy, were associated with prognosis. Based on the subsequent multivariate Cox regression analysis, patients who received NART or ART, ≤65 years of age, marital status (married), lower tumor grade, partial gastrectomy, ELNs ≥ 15, intestinal type, and chemotherapy were protective factors, whereas advanced TNM stage was a significant risk factor (**Figure 6**; **Table 2**). A superior risk threshold probability of 5% to 22% was observed in the DCA of the net benefit when compared with the baseline (**Figure 7**).

**Table 2:** Univariate and multivariate Cox regression analyses of overall survival.

Characteristics	Univariate		Multivariate	
	HR (95%CI)	*p-*Value	HR (95%CI)	*p-*Value
**RT sequence (vs. ART)**				
NART	0.96 (0.87–1.06)	0.394	1.21 (1.09–1.35)	<0.001
None	1.37 (1.25–1.51)	<0.001	1.21 (1.10–1.36)	<0.001
**Sex (vs. female)**	0.99 (0.90–1.09)	0.872		
**Age (vs. ≤65 years)**	1.40 (1.30–1.51)	<0.001	1.32 (1.22–1.43)	<0.001
**Marital status (vs. married)**				
Single	1.04 (0.92–1.18)	0.532	1.07 (0.95–1.22)	0.257
Others	1.24 (1.13–1.36)	<0.001	1.16 (1.06–1.28)	0.002
**Race (vs. Black)**				
White	1.00 (0.83–1.20)	0.969		
Others	0.87 (0.70–1.09)	0.219		
**Grade (vs. I/**II**)**	1.28 (1.18–1.39)	<0.001	1.21 (1.11–1.32)	<0.001
**Histological type (vs. diffuse type)**				
Intestinal-type gastric adenocarcinoma	0.83 (0.75–0.92)	0.001	0.88 (0.79–0.98)	0.019
Others	0.88 (0.75–1.03)	0.100	0.89 (0.76–1.04)	0.149
**Surgery (vs. gastrectomy, NOS)**				
Near total/total gastrectomy	0.91 (0.83–1.01)	0.069	0.92 (0.83–1.01)	0.083
Partial gastrectomy	0.83 (0.71–0.97)	0.018	0.79 (0.67–0.92)	0.003
**ELNs (vs. <15)**	0.82 (0.76–0.88)	<0.001	0.75 (0.70–0.81)	<0.001
**TNM stage (vs.** T_1–2_N^+^ **)**				
T_3–4_N^−^	0.56 (0.50–0.62)	0.093	1.11 (1.04–1.18)	0.001
T_3–4_N^+^	1.08 (0.99–1.17)	<0.001	1.71 (1.57–1.85)	<0.001
**Chemotherapy (vs. no)**	0.57 (0.52–0.62)	<0.001	0.65 (0.58–0.72)	<0.001

NART, neoadjuvant radiotherapy; RT, radiotherapy; ART, adjuvant radiotherapy; ELN, number of examined lymph node.

**Figure 8:**
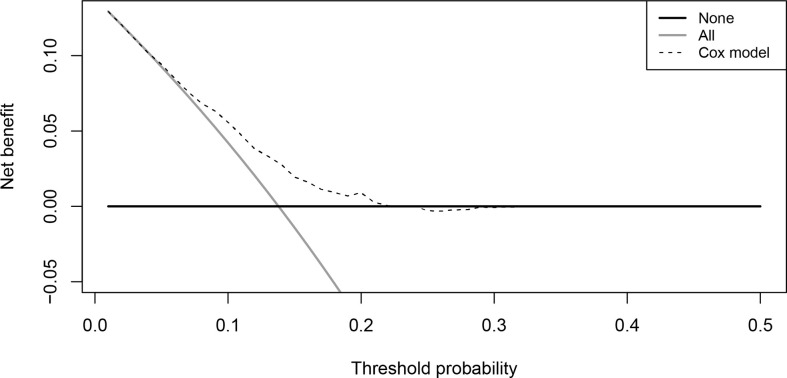
Calibration curve of Cox model.

The Cox model well-fitted the observed data, as shown by the calibration plot, in which the calibration curve overlapped with the diagonal of the reference line of perfect calibration (**Figure 8**).

## Discussion

Previously, patients with gastric cancer patients and those with GEJA have been considered the same population, given that both patient groups received identical treatments. Subsequently, growing data have suggested that GEJA should be considered an independent disease (15, 16). Accordingly, in the present study, we aimed to identify a precise treatment strategy for patients with stage II–III GEJA. Herein, we employed a large population-based cohort that included representative demographic, clinical, and biological characteristics to examine the prognostic significance of NART and ART in patients with stage II–III GEJA. Our study evaluated the differences in survival in patients who underwent NART or ART, grouped by TNM stage and histological type. These results may be valuable for clinicians in screening potential candidates who would benefit from NART or ART.

Complete resection of the primary tumor regional lymph nodes is pivotal for improving the prognosis of patients with resectable GEJA. GEJA has a distinct lymph node drainage pathway in esophageal and gastric cancers (15). The extent of lymph node resection remains debatable, posing a challenge for the surgeon to ensure R0 resection in patients with stage II–III GEJA. Historically, RT has been the standard adjuvant treatment for gastric cancer, based on reported randomized controlled trials (3). The Korean ARTIST-1 study has demonstrated that adjuvant chemoradiotherapy can improve disease-free survival when compared with adjuvant chemotherapy alone in gastric/gastroesophageal junction adenocarcinomas (16). Kim et al. (17) have shown that chemoradiation could improve locoregional recurrence-free survival in stage III gastric cancer treated with R0 gastrectomy and D2 lymph node dissection when compared with chemotherapy. Few studies have investigated the effect of ART in patients with stage II–III GEJA. Herein, we found that ART afforded significant survival benefits in patients with T_1–2_N^+^ and T_3–4_N^+^ tumors. Thus, we speculated that lymph node status might play a predominant role in mediating the positive effects of ART, and the results of the present study corroborate those of previous studies (18). However, whether a patient undergoes complete postoperative RT should be determined based on the patient’s physical condition, such as chronic underlying diseases, limited functional capacity, and poor clinical condition.

Considering the low completion rate of ART, it is of considerable importance to explore the impact of NART in stages II–III GEJA. NART can help reduce the preoperative stage and improve the likelihood of R0 resection. Moreover, patients exhibit better tolerance to the full course of NART than to ART. Based on the findings of our study, survival advantages afforded by NART could be noted in patients with T_3–4_N^+^ and T_3–4_ with intestinal type, which was similar to the findings of a previous study (14), which failed to consider the implications of ART in GEJA. According to Shridhar et al., NART could improve survival rates in patients with lymph node involvement; however, the authors failed to perform subgroup analysis by T stage stratification (19). In the present study, NART did not afford survival benefits in patients with T_3–4_N^−^ and T_1–2_N^+^ stages. This finding might indicate that benefits conferred by NART may fail to significantly surpass NART-induced side effects in patients with a low primary tumor burden or those without regional lymph node metastasis (7). In brief, both NART and ART could provide additional survival benefits in the T_3–4_N^+^ GEJA subgroup. For T_3–4_N^−^ and T_1–2_N^+^ stages, the application of NART needs to be further explored, considering new RT techniques, different dosages, and fractionation schemes.

In the prognosis analysis, multivariate Cox regression identified that receiving NART or ART, ≤65 years of age, married, lower tumor grade, partial gastrectomy, ELNs ≥ 15, intestinal type, and chemotherapy were protective factors, whereas an advanced TNM stage was a significant risk factor. A low number of ELNs was associated with decreased overall survival, consistent with previous studies (18, 19). In addition, our study revealed that being married could be a protective factor for better outcomes. Wang et al. (20) suggested that married patients potentially experienced less distress and depression after a cancer diagnosis and received spousal encouragement, enabling them to accept treatment better. Moreover, diffuse-type gastric adenocarcinoma exhibited an overall poorer prognosis than the intestinal type, consistent with a study by Tang et al. (21).

However, the limitations of this study need to be addressed. First, this study lacked accurate information regarding RT dosage, irradiation site, and RT techniques. Second, surgical margins are a critical factor affecting patient outcomes; we could not obtain this information from the SEER database. Finally, we failed to verify the results externally by assessing non-database cases. Therefore, the conclusions of this study require further validation.

## Conclusion

Both NART and ART can prolong the survival rate of patients with stage II–III GEJA. ART may afford survival benefits in patients presenting the T_1–2_N^+^ stage. Patients in stage T_3–4_ with intestinal type could benefit from NART. Moreover, NART and ART could positively impact the prognosis of patients with T_3–4_N^+^ GEJA.

## Data availability statement

Publicly available datasets were analyzed in this study. This data can be found here: https://seer.cancer.gov/.

## Author contributions

YL had full access to all of the data in the study and take responsibility for the integrity of the data and the accuracy of the data analysis. Concept and design: ZZ, YP and YZ. Acquisition, analysis, or interpretation of data: ZZ, SL, WZ and XZ. Drafting of the manuscript: ZZ, YJZ, BL, JM and ML. Statistical analysis: ZZ, YP, YZ, SL, WZ and XZ. All authors contributed to the article and approved the submitted version.

## Conflict of interest

The authors declare that the research was conducted in the absence of any commercial or financial relationships that could be construed as a potential conflict of interest.

## Publisher’s note

All claims expressed in this article are solely those of the authors and do not necessarily represent those of their affiliated organizations, or those of the publisher, the editors and the reviewers. Any product that may be evaluated in this article, or claim that may be made by its manufacturer, is not guaranteed or endorsed by the publisher.
